# Secondary benefits of residential treatment camp for parents of adolescents with problematic internet use

**DOI:** 10.1002/pcn5.70389

**Published:** 2026-08-07

**Authors:** Hidenori Ohnishi, Shun Umino, Hiromi Suzuki, Nobuyuki Miyatake, Yuu Nakamura

**Affiliations:** ^1^ Sanko Hospital Takamatsu Kagawa Japan; ^2^ Department of Neuropsychiatry, Faculty of Medicine Kagawa University Miki Kagawa Japan; ^3^ Department of Hygiene, Faculty of Medicine Kagawa University Miki Kagawa Japan

**Keywords:** intervention, parents, problematic internet use, residential treatment camp, text mining

## Abstract

**Aim:**

Residential treatment camps for problematic internet use (PIU) may influence adolescents' behaviors and parental mental health and parenting attitudes. This secondary analysis examined the effects of a residential treatment camp on parents of adolescents with PIU.

**Methods:**

This secondary analysis included 20 parents (3 fathers and 17 mothers) of adolescents who participated in a residential treatment camp for PIU. Parents were assessed employing the General Health Questionnaire‐12 (GHQ‐12), Positive and Negative Parenting Scale (PNPS), and State‐Trait Anxiety Inventory Form JYZ (STAI‐JYZ). Qualitative analysis was conducted using free‐response questionnaires regarding perceptions of adolescents' internet and gaming use. These evaluations were performed at baseline, after the intervention, and at the 3‐month follow‐up.

**Results:**

The GHQ‐12 scores demonstrated significant changes throughout the study period. They improved significantly after the intervention but deteriorated significantly at follow‐up compared with after the intervention. No significant changes were identified in STAI‐JYZ scores or PNPS positive parenting scores. The PNPS (negative attitude) showed significant changes between baseline and follow‐up and between after the intervention and follow‐up. Text mining results showed a transition from restrictive terms such as “protect” and “reduce” at baseline to autonomy‐related terms including “myself,” “decide,” and “freedom” after the intervention and at follow‐up.

**Conclusion:**

A residential treatment camp intervention for adolescents with PIU did not produce any significant long‐term effects on their parents' mental health. However, text mining results suggested a shift in parental perspectives from restrictive control toward greater respect for adolescents' autonomy.

## INTRODUCTION

Excessive internet and gaming use has emerged as a major public health concern because of its adverse effects on physical health, mental health, and social functioning of young people.^1^, ^2^, ^3^ In Japan, problematic internet use (PIU) is highly prevalent and has been linked to sleep disturbances, mental health problems, and social withdrawal.^4^, ^5^, ^6^ The problem has been further exacerbated by increasingly early and intensive digital exposure, highlighting the urgent need for effective countermeasures.^7^ In response, Kagawa Prefecture in Japan established the “Internet and Game Addiction Countermeasures Ordinance” in 2020. However, the ordinance faced criticism regarding political intervention in family affairs and children's rights, and alternative countermeasures have not yet been sufficiently implemented.^6^

Various interventions for PIU, including cognitive‐behavioral therapy (CBT) and family therapy, have been introduced.^8^ More recently, residential treatment camps have attracted considerable attention. Previous studies have demonstrated that participation in such camps may reduce adolescents' internet dependency.^9^ PIU is known to impose substantial psychological burdens on young people and their families, particularly parents.^10^ In addition, parental attitudes and psychological states are important factors influencing children's prognoses.^11^, ^12^, ^13^, ^14^, ^15^ Nevertheless, longitudinal evidence regarding the effects of residential treatment camps on parents themselves remains extremely limited. To date, the only longitudinal study examining the effects of treatment camps on parents themselves is the 7‐day “Siriraj Therapeutic Residential Camps” for adolescents with PIU. Although the study followed families for 6 months, the evaluation focused primarily on adolescents' PIU severity, and changes in parental psychological outcomes were not assessed.^16^

The present study is a secondary analysis of the effectiveness of a residential treatment camp conducted in Kagawa Prefecture, as reported by Suzuki et al.^17^ In this study, we aimed to examine the effects of residential treatment camps on parents' mental health, parenting attitudes, and perceptions of their children using quantitative and qualitative indicators. To date, most research on treatment camp‐based interventions has mainly focused on changes in adolescents' PIU symptoms, whereas psychological and cognitive changes in parents have received limited attention. Furthermore, no previous study has longitudinally examined the changes in parents' mental health and parenting attitudes by integrating quantitative and qualitative data. Given the limited prior evidence in this area, the present study was exploratory rather than confirmatory, and no formal a priori hypotheses were specified.

## METHODS

### Participants

A total of 20 parents (17 mothers and 3 fathers) of the eligible 24 adolescent participants were included in this secondary longitudinal observational study: (1) their children participated in a 6‐night, 7‐day residential treatment camp designed to support recovery from PIU; (2) they attended the orientation and selection meeting and completed the baseline questionnaire; (3) when multiple children from the same family participated in the camp, only the questionnaire for the child with the lowest randomly assigned participant number was included to avoid duplicate parental responses; and (4) they provided written informed consent (Table 1).

**Table 1:** Characteristics of enrolled participants.

	*n*	%
Number of participants	20	100
Mother	17	85
Father	3	15
Child grade level		
Elementary school (Years 5–6)	11	55
Junior high school (Years 7–9)	7	35
High school (Years 10–12)	2	10
Parent age group		
30s	6	30
40s	12	60
50s	2	10

The study protocol was approved by the Ethics Committee of the Faculty of Medicine, Kagawa University, Kagawa, Japan (approval number: 2024‐023, approval date: 2024/06/21).

### Intervention for parents

The parent intervention program, implemented as part of the residential treatment camp, consisted of psychoeducation and family participation sessions. Psychoeducational sessions for parents were held during the orientation on the first and final days of the camp. These sessions aimed to improve parents' understanding of PIU, facilitate conceptualization of the recovery process based on the transtheoretical stages‐of‐change model,^18^ and promote appropriate family engagement strategies within the home environment.

Specifically, parents were encouraged to view their children's internet and gaming not solely as problematic behaviors but as part of an ongoing behavioral change process. The program also emphasized positive parenting approaches grounded in CBT principles. In addition, core concepts of positive communication and motivational interviewing were introduced,^19^ with a focus on empathetic communication that supports children's intrinsic motivation rather than directive or controlling interactions. This psychoeducational program included lectures and experience‐sharing sessions involving individuals recovering from addiction and their families, as well as parent group discussions. These activities were designed to encourage reflection on parenting attitudes and perceptions toward their children. A detailed, session‐by‐session outline of the parent intervention program is provided in Supporting Information S1: Table 1.

### Measurements

Questionnaire surveys were administered to the same parents at three time points: during orientation in July 2024 (baseline), on the final day of the main camp in August 2024 (post‐intervention), and during the follow‐up camp conducted 3 months after completion of the main camp in November 2024 (3‐month follow‐up). The overall study design and data collection procedure have been described previously.^17^

The General Health Questionnaire‐12 (GHQ‐12), Positive and Negative Parenting Scale (PNPS), and State‐Trait Anxiety Inventory Form JYZ (STAI‐JYZ) were employed to assess the parents' mental health status and parenting attitudes. In addition, qualitative data were collected using an open‐ended questionnaire asking, “What are your thoughts on how your children use the internet and video games?”

### Mental status

The GHQ‐12 is a 12‐item instrument designed to assess nonspecific psychological distress, including symptoms of depression and anxiety.^20^ It has been widely used in non‐psychiatric medical settings, occupational and industrial health settings, educational institutions such as schools and universities, and community‐based populations, including parent groups.^21^ Previous studies have confirmed its good internal consistency and construct validity.^22^

### Parenting style

The PNPS is a 24‐item self‐report instrument that evaluates parenting behaviors from positive and negative perspectives. Its validity and reliability have been established in Japan.^23^, ^24^ Positive parenting reflects warmth, responsiveness, and promotion of autonomy, whereas negative parenting reflects overprotection, strict discipline, or inconsistency. The STAI‐JYZ consists of 40 items and is evenly divided into two subscales assessing state anxiety and trait anxiety.^25^, ^26^ State anxiety reflects a temporary response to situational factors, whereas trait anxiety represents a relatively stable tendency toward anxiety.

### Quantitative analysis

Statistical analyses were performed using longitudinally collected data. Given the small sample size and non‐normal data distribution, continuous variables are presented as medians and interquartile ranges (IQRs). Differences across time points were analyzed using the Friedman test followed by the Wilcoxon signed‐rank test with Bonferroni correction for post hoc comparisons. Statistical significance was defined as *p* < 0.05 for the Friedman test and *p* < 0.017 (0.05/3) for the Wilcoxon signed‐rank test. Analyses were conducted using data from participants with valid values for each outcome measure, excluding those with missing values or invalid responses. In addition, effect sizes (*r*) were calculated as *Z*/√*N*, where *N* is the total number of observations (number of pairs × 2), to aid interpretation of the magnitude of the observed changes given the small sample size. Statistical analyses were conducted using JMP Pro 18 software (SAS Institute Inc., Cary, NC, USA).

### Qualitative analysis (text mining)

Qualitative analysis was performed using the text‐mining software KH Coder,^27^ an open‐source platform widely used for quantitative content analysis and text mining in public health, nursing, psychology, and social science research. The methodological framework of KH Coder has been described previously by Higuchi using a two‐step quantitative content analysis approach.^28^, ^29^ First, all open‐ended data were digitized and anonymized prior to morphological analysis, which segmented sentences into word units. Changes in vocabulary size and word content across time points were subsequently compared. Co‐occurrence network analysis was conducted to visualize relationships among frequently co‐occurring words. In these networks, words were represented as nodes, and co‐occurrence relationships within the same sentence were represented as edges. Node size indicated word frequency, whereas edge thickness reflected the strength of the co‐occurrence between words.^30^

Qualitative analysis was used as an exploratory approach and to complement and facilitate interpretation of the quantitative findings.

## RESULTS

### Participants

At baseline, 20 parents completed the initial assessment. However, some participants did not complete the follow‐up evaluation during the follow‐up period. Furthermore, owing to missing or invalid responses, the number of participants included in each analysis varied according to the assessment measure (Table 1).

### Quantitative analysis

**Figure 1:**
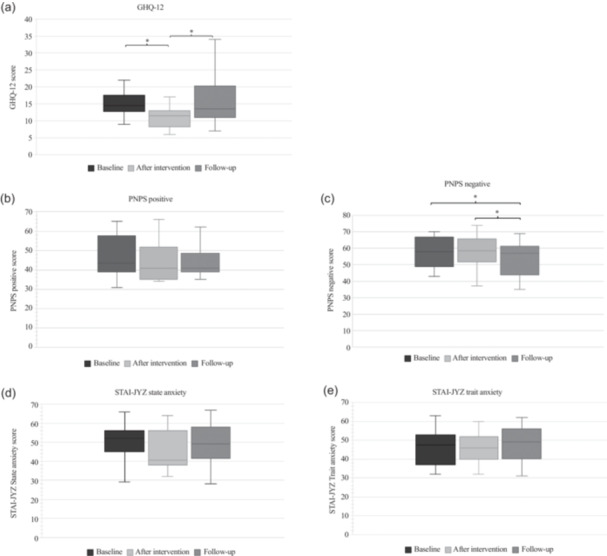
Changes in General Health Questionnaire‐12 (GHQ‐12), Positive and Negative Parenting Scale (PNPS), and State‐Trait Anxiety Inventory (STAI) scores at baseline, after intervention, and at follow‐up. (a) GHQ‐12, (b) PNPS positive, (c) PNPS negative, (d) STAI state anxiety, and (e) STAI trait anxiety. *p*‐Values were computed using the Friedman test, followed by Wilcoxon signed‐rank tests with Bonferroni correction.

The GHQ‐12 scores demonstrated significant changes across the three time points. Because higher GHQ‐12 scores indicate greater psychological distress, lower scores reflect better mental health. Multiple comparisons showed significantly lower scores after the intervention than at baseline, indicating improvement in parental mental health. However, scores at follow‐up were significantly higher than those after the intervention and returned to levels comparable to baseline (Table 2, Figure 1a). No significant changes were identified in the PNPS positive parenting attitude scores across the three time points (Table 2, Figure 1b). In contrast, the PNPS negative parenting attitude scores revealed significant changes across the three time points. Multiple comparisons analysis demonstrated significant reductions from baseline to follow‐up and from post‐intervention to follow‐up, indicating improvement in negative parenting attitudes (Table 2, Figure 1c). State and trait anxiety scores assessed using the STAI‐JYZ showed no significant changes across the three time points (Table 2, Figure 1d,e).

**Table 2:** Changes in parameters with a residential treatment camp.

	Baseline (A)	After intervention (B)	Follow‐up (C)	Change (A vs. B)		Change (A vs. C)		Change (B vs. C)	
	Median (interquartile range [IQR])	Median (IQR)	Median (IQR)	Median (IQR)	*p*‐Value	Median (IQR)	*p*‐Value	Median (IQR)	*p*‐Value
General Health Questionnaire‐12	*n* = 20	*n* = 20	*n* = 18	−4.5 (−6.3 to 0.0)	*r* = −0.54	−1.5 (−4.3 to 2.3)	*r* = −0.02	3.0 (0.3–5.0)	*r* = 0.47
	14.5 (12.8–17.5)	11.5 (8.8–13.0)	13.5 (11.3–19.3)		**0.001**		0.454		**0.001**
Positive and Negative Parenting Scale	*n* = 17	*n* = 17	*n* = 14	−2.0 (–8.0 to 1.0)	*r* = −0.32	−2.0 (−9.0 to 0.0)	*r* = −0.29	2.0 (–11.8 to 4.5)	*r* = 0.05
Positive attitude	42.0 (39.0–52.0)	39.0 (35.0–51.0)	41.0 (39.3–46.5)		0.151		0.202		0.506
Positive and Negative Parenting Scale	*n* = 17	*n* = 17	*n* = 14	−1.0 (–4.0 to 0.0)	*r* = −0.06	−5.5 (−9.3 to 0.8)	*r* = −0.74	–5.0 (–8.0 to 0.0)	*r* = −0.62
Negative attitude	56.0 (48.0–63.0)	56.0 (48.0–62.0)	57.0 (45.3–60.0)		0.679		**0.002**		**0.007**
State‐Trait Anxiety Inventory‐JYZ	*n* = 17	*n* = 17	*n* = 16	−6.0 (−15.0 to 7.0)	*r* = −0.29	0.0 (−7.3 to 6.0)	*r* = −0.10	2.5 (–3.0 to 9.0)	*r* = 0.26
State anxiety	51.0 (45.0–56.0)	40.0 (38.0–53.0)	49.0 (44.5–56.0)		0.155		0.726		0.236
State‐Trait Anxiety Inventory‐JYZ	*n* = 17	*n* = 17	*n* = 16	−1.0 (−3.0 to 3.0)	*r* = −0.04	2.5 (−0.8 to 4.3)	*r* = 0.13	2.5 (–2.3 to 4.5)	*r* = 0.16
Trait anxiety	46.0 (36.0–52.0)	46.0 (39.0–52.0)	49.0 (40.8–56.0)		0.500		0.364		0.411

*Note*: *r*, effect size calculated as *Z*/√*N*, where *N* is the total number of observations (number of pairs × 2). The sign of *r* indicates the direction of change. According to Cohen's criteria, *r* values of approximately 0.1, 0.3, and 0.5 indicate small, medium, and large effects, respectively. Bold values indicate statistically significant differences.

### Qualitative analysis

The total number of extracted words from the open‐ended responses was 284 at baseline, 373 after the intervention, and 852 at follow‐up. Co‐occurrence network analysis identified “protect” and “reduce” as characteristic terms at baseline, “myself” and “decide” after the intervention, and “freedom” at follow‐up. Furthermore, the diversity of vocabulary and the complexity of inter‐word relationships increased after the intervention and at follow‐up, resulting in more elaborate co‐occurrence network structures (Supporting Information S2: Figure 1, Supporting Information S3: Figure 2, and Supporting Information S4: Figure 3).

## DISCUSSION

This secondary analysis of a residential treatment camp for adolescents with PIU in Kagawa Prefecture, Japan, longitudinally examined intervention‐associated changes in parents using quantitative measures and qualitative text mining. Although the original study reported improvements in adolescents' PIU severity and mental health,^17^ the improvement in parental mental health assessed by the GHQ‐12 was only transient and returned to near‐baseline levels at follow‐up. In contrast, negative parenting attitudes decreased, and the language parents used to describe their children's internet and gaming use shifted over the same period. We interpret these observations as preliminary and hypothesis‐generating: the camp may be associated more with changes in how parents describe and relate to their children than with durable relief of their own psychological distress, although this interpretation requires confirmation in controlled studies.

The transient improvement in GHQ‐12 scores may indicate temporary psychological relief during the intervention period, followed by the re‐emergence of parental stress after returning to daily life. Parents of children with PIU often experience chronic psychological stress in their everyday lives,^10^ which may not be sufficiently alleviated by short‐term intensive interventions alone. Similar findings have been reported in studies of child‐focused psychiatric interventions. For example, maternal anxiety and depressive symptoms following inpatient family treatment remained elevated compared with community standards at the 3‐ and 12‐month follow‐up assessments.^31^ In addition, a meta‐analysis of parent training programs demonstrated that moderate post‐intervention effects on parental behavior and perceptions were substantially attenuated within 1 year, leading the authors to emphasize the importance of continuing‐care approaches rather than short‐term intensive interventions.^32^ In contrast, intervention effects among adolescents themselves may be more sustained,^9^ which may suggest that residential treatment could exert different temporal effects on adolescents and parents. The transient nature of this change was also reflected in the effect sizes: the improvement from baseline to after the intervention was large (*r* = −0.54), whereas the deterioration from after the intervention to follow‐up was moderate to large (*r* = 0.47), and the difference between baseline and follow‐up was negligible (*r* = −0.02).

Negative parenting attitudes decreased significantly from baseline to follow‐up, whereas positive attitudes remained unchanged. This selective pattern, characterized by reductions in dysfunctional parenting without corresponding increases in positive parenting behaviors, is consistent with evidence suggesting that negative interactions exert disproportionately strong effects on close interpersonal relationships.^33^ It also aligns with reports indicating that family‐oriented interventions often produce domain‐specific rather than uniform changes in parenting behaviors.^31^ One possible explanation for these findings may involve the transtheoretical model and motivational interviewing principles incorporated into the psychoeducational program. From the perspective of the transtheoretical model,^18^ a child's participation in the camp may be regarded as a behavioral expression of an internal shift from pre‐contemplation or contemplation toward action. Miller and Rose further argue that recognizing ambivalence and eliciting “change talk” are key mechanisms underlying behavioral change.^19^ Through psychoeducation, parents may have become more aware of their children's ambivalent psychological state, simultaneously recognizing the need for change while continuing internet and gaming behaviors, and consequently adopted less confrontational responses, plausibly contributing to the observed reduction in negative parenting attitudes. Collectively, these findings suggest that the camp's effects on parenting may primarily reduce maladaptive interactions rather than directly enhance positive parenting behaviors. Notably, negative parenting attitudes changed little between baseline and the end of the camp (*r* = −0.06) but showed a large reduction by follow‐up (baseline vs. follow‐up, *r* = −0.74; after intervention vs. follow‐up, *r* = −0.62). This suggests that the change did not occur during the camp itself but developed over the subsequent 3 months, possibly as parents applied the psychoeducational content in daily life. This pattern also contrasts with that of parental psychological distress (GHQ‐12), which improved markedly during the camp but had returned to baseline by follow‐up. Although this dissociation should be interpreted cautiously given the small sample and the absence of a control group, it raises the possibility that the camp's association with parental distress and with parenting attitudes may operate through partly independent and differently timed processes.

The text mining results documented changes in the words parents used and in their co‐occurrence patterns, rather than directly measuring changes in cognition or family relationships. At baseline, restrictive terms such as “protect” and “reduce” were prominent, whereas autonomy‐related terms including “myself,” “decide,” and “freedom” became increasingly characteristic after the intervention and at follow‐up. In addition, the increase in the number of extracted words and the greater complexity of the co‐occurrence networks suggest that parents' narratives evolved from descriptions focused primarily on internet use behaviors to more integrated perspectives involving judgment, prioritization, and self‐regulation. This linguistic change is consistent with, but does not by itself demonstrate, a shift from a more control‐oriented toward a more autonomy‐supportive way of describing children's internet and gaming use; such a shift might in turn create a relational context relevant to adolescents' motivation, as discussed in the context of motivational interviewing.^19^ The methodological value of text mining as a complementary qualitative approach in health research has been increasingly recognized. KH Coder‐based co‐occurrence network analysis has been applied in awareness surveys among healthcare workers and medical and healthcare students regarding infectious diseases, such as syphilis, acquired immunodeficiency syndrome (AIDS), and coronavirus disease 2019 (COVID‐19), demonstrating the utility of quantitative text mining for analyzing free‐text responses that are difficult to evaluate using conventional approaches.^34^, ^35^, ^36^ Similarly, text mining analyses of nursing care records and hospital fall incident reports have identified latent thematic structures that may not be readily captured through traditional content analysis methods.^37^, ^38^ Furthermore, a systematic review of text‐mining applications in psychiatry supported the utility of this approach for identifying semantic features associated with psychological states and extracting patients' perspectives from narrative data.^39^ In the present study, the observed changes in parents' vocabulary may similarly reflect a broadening of their perspectives on their children's internet and gaming use. Whether such linguistic changes correspond to cognitive or relational transformation within the family system cannot be determined from the present data and remains a hypothesis to be tested in future research.

This study has some limitations. First, the study sample was relatively small, especially for fathers, and the study employed a single‐group pre‐post design without a control group. Second, participants were limited to parents who were concerned about PIU and voluntarily enrolled their children in a residential treatment camp. Therefore, the enrolled participants were thought to be more health‐conscious than the general population. Third, the follow‐up period was relatively short, and longer term investigations are required. Fourth, parenting attitudes were evaluated using self‐report measures rather than objective behavioral assessments. Thus, the observed changes may partly reflect reporting bias or altered parental perceptions, rather than actual behavioral changes.

Future research should address these limitations in several ways. First, larger scale studies including an appropriate control group of parents whose adolescents did not participate in residential treatment camps are required to clarify the magnitude of the intervention‐specific effect. Second, longer follow‐up periods (e.g., 12 months or longer) would help determine whether the observed reductions in negative parenting attitudes and the shift toward autonomy‐supportive cognition result in sustained improvements in family functioning and adolescent outcomes. Third, mechanistic investigations examining the relative contributions of specific intervention components, including psychoeducation, family exchange meetings, lived‐experience testimonies, or exposure to motivational interviewing, may help optimize and refine family‐oriented camp programs for PIU. In addition, future studies could examine whether a lexical shift toward autonomy‐related language constitutes a measurable marker of autonomy‐supportive parenting and employ dyadic analyses linking changes in parental language to adolescent PIU outcomes.

In summary, this secondary analysis evaluated the effects of a residential treatment camp for adolescents with PIU on parental outcomes. Although no sustained long‐term improvement in parental mental health was observed, reductions in negative parenting attitudes and cognitive shifts toward greater respect for adolescents' autonomy were identified. These findings suggest that residential treatment camps may primarily influence how parents understand and interact with their children, rather than directly improving parental psychological distress.

## AUTHOR CONTRIBUTIONS

Hidenori Ohnishi conducted the survey, collected and analyzed the data, and drafted the manuscript. Shun Umino planned and operated the treatment camp and conducted the survey. Hiromi Suzuki analyzed the data, evaluating the therapeutic effects on children. Nobuyuki Miyatake analyzed the data and critically reviewed the manuscript. Yuu Nakamura supervised the study and provided scientific advice. All authors reviewed and approved the final manuscript.

## CONFLICT OF INTEREST STATEMENT

The authors declare no conflicts of interest.

## ETHICS APPROVAL STATEMENT

The study protocol was approved by the Ethics Committee of the Faculty of Medicine, Kagawa University, Kagawa, Japan (approval number: 2024‐023, approval date: 2024/06/21).

## PATIENT CONSENT STATEMENT

All participants provided written informed consent prior to participation. The informed consent process included an explanation that the study results might be published in academic journals in a form that does not allow the identification of individuals. This article does not contain any individually identifiable information.

## CLINICAL TRIAL REGISTRATION

Not applicable.

## Supporting information

Supplementary_Table_S1.

Supplementary_Figure_S1.

Supplementary_Figure_S2.

Supplementary_Figure_S3.

## Data Availability

The data that support the findings of this study are available from the corresponding author upon reasonable request. Research data are not publicly available due to confidentiality restrictions.
