# Supplementary material for: Secondary benefits of residential treatment camp for parents of adolescents with problematic internet use
Source: PCN Rep. 2026 Aug 7;5(3):e70389. doi: 10.1002/pcn5.70389 (PMC13448770; doi:10.1002/pcn5.70389)
Supplement: Supplementary file 1 [file PCN5-5-e70389-s002.docx]

| Supplementary Table S1. Outline of the parent intervention program of the residential treatment camp. | | |
| --- | --- | --- |
| Session | Activity | Content |
| Orientation (July 2024) | Group work: "Sharing what we do not understand," "Role play," "Five steps of active listening" | Parents were divided into small groups to discuss concerns regarding their children. Based on these discussions, communication scenarios were developed and role-play was conducted. |
|  | Lecture: "Internet and gaming dependency" | A lecture was delivered on adolescence as a preparatory stage for addiction, prevention approaches to internet and gaming dependency, and the iSwing program using text-based educational material. |
| First day of camp (August 2024) | Parent–child icebreaker: "Telepathy" | Parents and children worked in pairs and answered the facilitator's questions simultaneously, aligning their responses with their partner's. |
|  | Group work: "Expanding the variety of praise" | In pairs, one parent described recent behavior of their child while the other listened, identified strengths, and verbalized them. Participants alternated roles repeatedly. |
|  | Parent–child guest lecture | Individuals who had recovered from internet and gaming dependency, together with their family members, shared lived experiences and recovery narratives. |
|  | Practice: "Conveying feelings (creating an appreciation certificate)" | Parents created an "award certificate" highlighting and affirming positive characteristics of their children. |
| Final day of camp (August 2024) | Lecture: "How does change occur?" | The program director delivered a lecture using practical examples to explain the Transtheoretical Model and motivational interviewing in the context of adolescents’ behavioral change and family responses. |
|  | Group work: "Learning positive ways of engaging" | Parents discussed their impressions of the lecture in small groups and explored strategies for balanced and constructive parent–child communication. |
|  | Child presentation session | Parents listened to each child's presentation. |
| Follow-up camp (November 2024) | Icebreaker: "Newspaper rock-paper-scissors" | Cooperative game with parent-child pairs. |
|  | Group work: "Could we engage positively?" / "Motivational interviewing" | Parents reflected on their interactions with their children during the 3 months after the camp. A brief overview of motivational interviewing was provided as a guidance for future engagement. |
|  | Child presentation session | Parents listened to each child's individual presentation. |

Supplementary Figure S1. Co-occurrence network analysis at baseline

Supplementary Figure S2. Co-occurrence network analysis after intervention

Supplementary Figure S3. Co-occurrence network analysis at follow-up
